# The potential role of T-cell metabolism-related molecules in chronic neuropathic pain after nerve injury: a narrative review

**DOI:** 10.3389/fimmu.2023.1107298

**Published:** 2023-05-17

**Authors:** Xiaoke Dou, Rui Chen, Juexi Yang, Maosha Dai, Junhao Long, Shujun Sun, Yun Lin

**Affiliations:** ^1^ Department of Anesthesiology, Union Hospital, Tongji Medical College, Huazhong University of Science and Technology, Wuhan, China; ^2^ Institute of Anesthesia and Critical Care Medicine, Union Hospital, Tongji Medical College, Huazhong University of Science and Technology, Wuhan, China; ^3^ Department of Pain, Union Hospital, Tongji Medical College, Huazhong University of Science and Technology, Wuhan, China

**Keywords:** chronic neuropathic pain, T cell, energy metabolism, P2X7R, PKM2, adiponectin

## Abstract

Neuropathic pain is a common type of chronic pain, primarily caused by peripheral nerve injury. Different T-cell subtypes play various roles in neuropathic pain caused by peripheral nerve damage. Peripheral nerve damage can lead to co-infiltration of neurons and other inflammatory cells, thereby altering the cellular microenvironment and affecting cellular metabolism. By elaborating on the above, we first relate chronic pain to T-cell energy metabolism. Then we summarize the molecules that have affected T-cell energy metabolism in the past five years and divide them into two categories. The first category could play a role in neuropathic pain, and we explain their roles in T-cell function and chronic pain, respectively. The second category has not yet been involved in neuropathic pain, and we focus on how they affect T-cell function by influencing T-cell metabolism. By discussing the above content, this review provides a reference for studying the direct relationship between chronic pain and T-cell metabolism and searching for potential therapeutic targets for the treatment of chronic pain on the level of T-cell energy metabolism.

## 1 Introduction

Chronic pain is defined as pain that lasts or recurs for three months or longer (1). The main clinical manifestations are spontaneous pain, hyperalgesia and allodynia (2), accompanied by existing or potential tissue damage. Neuropathic pain is the most common type of chronic pain, with a prevalence of over 80% (3) and the most comprehensive and established animal models (4). It is characterized as a chronic pain syndrome resulting from a lesion or disease affecting the sensory nervous system (5), comprising central and peripheral neuropathic pain (CNP and PNP) (6). PNP, which can occur after nerve injury, is the most common type of clinical neuropathic pain (7, 8).

The mechanism of neuropathic pain mainly involves peripheral sensitization and central sensitization. Peripheral sensitization refers to the phenomenon that the threshold of nociceptors is reduced due to nerve injury (9), while central sensitization is often manifested by the enhanced response of the pain loop in the central nervous system (CNS) (10). Infiltrated macrophages and T cells, as well as activated microglia and astrocytes, can release several pain regulators, contains proinflammatory cytokines, such as tumor necrosis factor-α (TNF-α), interleukin-1β (IL-1β), IL-17 and interferon-γ (IFN-γ), causing pain hypersensitivity (11–14). Particularly, T cells are able to infiltrate the dorsal root ganglion (DRG) and release the pro-inflammatory mediator leukocyte elastase (LE), leading to mechanical ectopic pain; and are involved in mechanical nociceptive hyperalgesia in the spinal cord, playing an important role in pain in the central and peripheral systems (15).

Cellular energy metabolism primarily relies on three nutrient types: carbohydrates, proteins, and fats. Normal cells break down proteins and fats into smaller molecules that enter the mitochondria to participate in the tricarboxylic acid (TCA) cycle. In contrast, glucose metabolism, a key aspect of carbohydrate metabolism, occurs in two distinct scenarios. Both conditions involve glycolysis, a series of biochemical reactions that convert glucose to pyruvate. Under aerobic conditions, pyruvate enters the mitochondrial TCA cycle for energy production, a process referred to as glucose oxidative phosphorylation (OXPHOS) or aerobic glycolysis. Under anaerobic conditions, pyruvate is converted to lactate in the cytoplasm while simultaneously generating energy, known as anaerobic glycolysis. However, tumor cells exhibit distinct energy metabolism compared to normal mature cells. Regardless of oxygen availability, tumor cells primarily rely on glucose consumption and lactate production for energy, a phenomenon known as the “Warburg effect” or aerobic glycolysis (16). Recent studies have demonstrated that activated T cells share similar energy metabolism with tumor cells, utilizing the “Warburg effect” as a critical energy source (17). To prevent ambiguity, we adopt the terms “anaerobic glycolysis” for lactate production in the cytoplasm and “glucose OXPHOS” for the process of glucose conversion to pyruvate followed by mitochondrial entry and OXPHOS. Moreover, the conversion of glucose to pyruvate is referred to as glycolysis. In recent years, the metabolic regulation of T-cell activity and function has become a focal point of research. This area of study has been widely explored in various diseases, such as oncology (18), metabolic syndrome (19), autoimmune diseases (20), and inflammatory diseases (21). While direct studies on the relationship between T-cell energy metabolism and neuropathic pain are relatively limited, there exist numerous molecules that play a role in T-cell metabolism and are implicated in the development of neuropathic pain.

The review first outlines the functions of different subpopulations of T cells in peripheral neuropathic pain arising from persistent nerve injury. Subsequently, it provides a comprehensive overview of the changes in the microenvironment caused by nerve injury and alterations in cellular energy metabolism that result from these changes. It also discusses the primary metabolic pathways adopted by T cells. We have focused on identifying the crucial metabolic molecules in T cells that have garnered attention in recent five years of research, categorizing them into two groups: (1) molecules of T-cell metabolism that participate in neuropathic pain, and (2) molecules of T-cell metabolism that have yet to play a role in neuropathic pain. These perspectives help to explore the critical role of T-cell metabolic molecules in the development and occurrence of neuropathic pain and provide potential targets for intervening in neuropathic pain.

## 2 T-cell functions in neuropathic pain

Different T cell subtypes play different roles. In the nerve injury-induced neuropathic pain model, CD4^+^ T cells infiltrate into the injured nerve, DRG, and spinal cord (22–26) and facilitate the transition from acute to chronic pain (25) and maintenance of chronic pain with different subpopulations acting distinctively (25, 27). Several studies have revealed that compared with mice from heterozygous litters, interferon-γ receptor 1 (IFN-γR1) deletion mutants and congenital athymic nude mice deficient in mature T cells show significantly less mechanical allodynia and thermal hyperalgesia after chronic constriction injury of the sciatic nerve (CCI) (28, 29). Corresponding transfer of T helper 1 cells (Th1) to nude mice enhances pain hypersensitivity in recipients to levels similar with the heterozygous donor rats (28, 29). In contrast, passive transfer of anti-inflammatory cytokine-producing Th2 into heterozygous rats significantly attenuates their pain hypersensitivity (29). Actually, the circulating levels of Th2-related IL-10 and IL-4 in patients with painless neuropathy are higher than those in patients with painful neuropathy and controls (30). These suggest that a Th2-mediated anti-inflammatory response may play a crucial role in regulating pain development. In a CCI model, IL-17A-positive T cells are detected by immunocytochemistry within the damaged nerve, and lack of Th17 is associated with reduced thermal nociceptive sensitization (26, 31). Depletion of regulatory T cells (Tregs) is achieved by injection of anti-CD25 antibody, and it is observed that Treg-depleted mice exhibit prolonged mechanical hypersensitivity (32). In studies of neuropathic pain resulting from peripheral nerve injury in mice, CD8^+^ T lymphocytes aggregate to a greater extent in the DRG and dorsal horn by migration after CCI, yet infiltrate mainly in the ventral horn after transection. These cells have been found to produce IL-10, which can relieve chronic pain (33), indicating that they may play a role in the resolution of chronic pain. However, it is worth noting that CD8^+^ T cells are not the only source of IL-10, as CD4^+^ T cells and macrophages are also capable of producing this cytokine (34).

## 3 T-cell energy metabolism in neuropathic pain

After peripheral nerve injury, various cells such as macrophages (35), glial cells (36), and lymphocytes (37) infiltrated the injured nerve, DRG, or spinal cord. These cells, along with the neurons, released numerous mediators that contributed to the alteration of the microenvironment in which they were located. These alterations induced a diverse range of effects on the cells in that environment, such as facilitating the conversion of inflammatory phenotypes (38) and triggering oxidative stress (39). Recent studies showed that metabolic abnormalities could occur in this microenvironment (40), which theoretically could modify neuropathic pain if rectified (41). Interestingly, metabolic abnormalities could also exist in T cells. Notably, in previous studies, it has been found that different types of T cells possess different metabolic patterns, and that their functions can be influenced by altering their metabolic processes.

### 3.1 Microenvironment changes due to nerve injury

In response to severe injurious stimuli, peripheral tissues and nerves may undergo a series of changes that induce alterations in the microenvironment surrounding the cells. For instance, nerve cells and other cells release a series of factors, causing to an inflammatory shift in the microenvironment. Specifically, following nerve lesions, neurons release reactive oxygen species/reactive nitrogen species (ROS/RNS) (42) and excess glutamate (43). Mast cells, neutrophils, and macrophages within the microenvironment subsequently release inflammatory factors such as adenosine triphosphate (ATP), bradykinin, prostaglandin E2, histamine, Serotonin (5-HT), IL-1β, IL-6, neural growth factor, and TNF-α, leading to peripheral inflammation. This inflammatory response further activates neurons to release pain-causing factors including substance P, calcitonin gene-related peptide (CGRP), neurokinin A, and nitrous oxide (NO), resulting in neurogenic pain (44). Sustained injurious stimuli lead to a prolonged inflammatory process, with lymphocytes releasing factors to create an “inflammatory soup” that lowers pain thresholds through peripheral sensitization due to inflammation-related changes (45).

In addition to eliciting inflammatory changes, the microenvironment experiences metabolic alterations. Functional neuroimaging studies reveal that glucose metabolism undergoes enhancement and glucose transporter 3 (GLUT3) protein expression increases in the medial prefrontal cortex (mPFC), which plays a pivotal role in neuropathic pain development (46). Classical antineuropathic pain therapeutics, such as gabapentin (47), and transcranial direct current stimulation (48), can mitigate pain responses by modulating glucose metabolism in the mPFC. The enhancement of glucose metabolism implies that there might be a temporary decrease in glucose within the microenvironment. These metabolic alterations result in the accumulation of various intermediates, including phosphoenolpyruvate (PEP) (41, 49) and lactate (41, 49, 50), which subsequently disturb the acid-base equilibrium in the microenvironment, driving a cascade of pathophysiological processes (41). Of note, astrocytes can produce L-lactate through the astrocyte-neuron lactate shuttle (ANLS), playing a crucial role in maintaining synaptic transmission (51, 52). Additionally, it has been shown to increase the concentration of glutamate after excitatory neuronal injury (53).

Alterations in the microenvironment could affect the cells within it and contribute to multiple pathological processes in neuropathic pain. For instance, the binding of ROS/RNS to receptors, such as Transient receptor potential cation channel subfamily M member 2 (TRPM2) expressed by glial cells and leukocytes, triggers the expression of pro-inflammatory mediators through the activation of mitogen-activated protein kinase (MAPK) and nuclear factor κB (NF-κB) (54, 55). Moreover, these extracellular changes can also result in alterations to the intracellular metabolism, which will be elaborated upon in the subsequent section.

### 3.2 Cellular metabolism alterations due to microenvironment changes

Due to microenvironment changes, various cellular metabolisms undergo certain alterations (40, 56). The accumulation of ROS/RNS by injury can impair mitochondria in the nociceptive pathway and induce mitochondrial dysfunction and can severely impair OXPHOS, resulting in metabolic disturbances (57), which has a significant impact on the mechanisms of neuropathic pain. It can occur in neurons, glial cells, and immune cells (55, 58), and restoring normal mitochondrial function can alleviate both the induction and persistence of pain (59). Targeting mitochondrial metabolism has also emerged as a potential therapeutic approach for neuroinflammation in progressive multiple sclerosis (60). Lactate can shuttle between glial cells and neurons, acting as one of the essential energy sources for neurons in the context of nerve injury, in order to maintain and promote pain transmission (51). Furthermore, Glutamate can be taken up by various cells and increase the metabolism of glutamine (41, 61, 62). It is noteworthy that, besides enhancing the metabolism of glutamate, glutamine also facilitates anaerobic glycolysis. Glutamate can be taken up by astrocytes *via* a sodium-dependent mechanism, which increases the intracellular sodium concentration, activates the sodium-potassium-ATPase on the cell membrane, promotes glucose uptake, and thus promotes anaerobic glycolysis (41). Furthermore, the presence of IFN-γ within the microenvironment has been implicated in potential modulatory effects on microglial cells (63). According to recent literature (63), brief exposure to β-amyloid alters the metabolic profile of microglia from OXPHOS to glycolysis by activating the mammalian target of rapamycin/hypoxia-inducible factor-1α (mTOR/HIF-1α) pathway. However, prolonged β-amyloid exposure reduces both glycolysis and OXPHOS, which impairs microglial responsiveness to harmful stimuli. In a murine model of Alzheimer’s disease, exogenous IFN-γ activates the mTOR pathway to promote glycolysis, which mitigates β-amyloid-induced microglial activation and ameliorates resultant neurofunctional deficits (63).

In particular, the metabolism of T cells could be influenced by alterations in the external environment, which in turn may affect a range of T cell functions. Current research has shown that activation of the T cell receptor (TCR) leads to enhanced uptake of GLUT1 on T cells (64). Following enhancement of T-cell glycolysis, the build-up of the metabolic intermediate phosphoenolpyruvate (PEP) can inhibit the endoplasmic reticulum (ER) calcium (Ca2+) channel, leading to impaired Ca2+ uptake by the ER Ca2+ store. This leads to a rise in cytoplasmic Ca2+ concentration, further activating the inflammatory pathway, driving T-cell differentiation towards a pro-inflammatory phenotype, and inducing the transcription of pro-inflammatory cytokines (41, 65). Lactate accumulation can enhance NF-κB pathway-mediated immune responses and inflammatory cascade reactions in Th17 (66). Excessive glutamate may contribute to enhanced glutamine metabolism in T cells, which is a potential outcome of altered T-cell energy metabolism.

### 3.3 T-cell function changes due to cellular metabolism alterations

**Figure 1:**
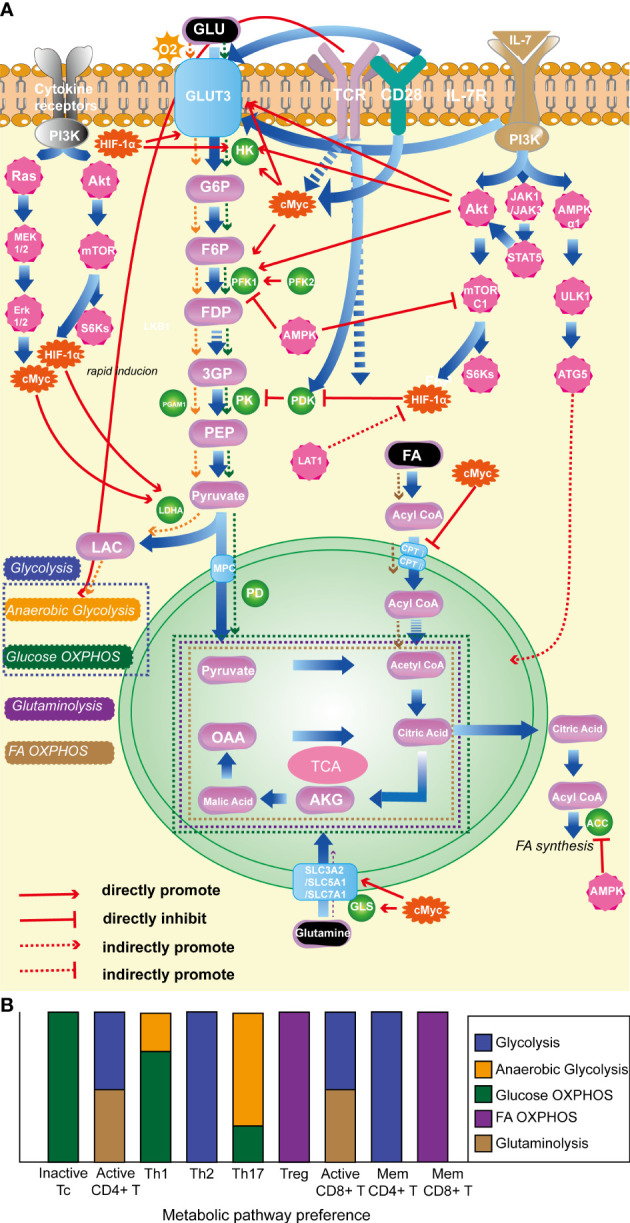
**(A)**. Classical molecules regulating T-cell energy metabolism. **(B)**. The energy metabolism preferences of T cells with different state and subtype. (1) HIF-1α: promotes glycolysis; inhibits OXPHOS of pyruvate; (2) c-Myc: enhances glycolysis; promotes glutamine catabolism. (3) AKT: foster glycolysis; (4) mTORC1: promotes glycolysis. (5) AMPK: prevent lipid synthesis; promote lipid oxidation; inhibit glycolysis. (6) TCR: promote glycolysis. (7) CD28: promote glycolysis. (8) IL-7R facilitates glycolysis; promotes OXPHOS. (9) LDHA: promotes glycolysis. HIF-1α, hypoxia-inducible factor-1α; mTORC1, the mammalian target of rapamycin complex 1; AMPK, AMP-activated protein kinase; TCR, T-cell receptor; IL-7R, interleukin-7 receptor; LDHA, lactate dehydrogenase.

It is noteworthy that various T-cell subtypes rely on distinct metabolic pathways, and their energy metabolism plays a pivotal role in their activation, differentiation, and effector functions (67). In a quiescent state, T cells primarily catabolize glucose and fatty acids (FA) *via* OXPHOS to maintain their basal cellular state and sustain their circulation in lymphoid tissues (17, 68). Correspondingly, when activated, CD4^+^ T cells and CD8^+^ cytotoxic T cells predominantly shift their metabolic program towards anaerobic glycolysis and glutaminolysis, allowing for the rapid generation of copious amounts of ATP and biosynthetic precursors necessary to sustain their activation and effector responses (69). Distinct metabolic programs are employed by different effector CD4^+^ T cell subtypes after activation. Specifically, Th1, Th2, and Th17 predominantly rely on glycolysis as their primary energy-generating pathway (70–72). Notwithstanding the fact that Th1 and Th17 cells predominantly rely on glucose as their principal fuel source and engage in glycolysis, their respective tendencies toward anaerobic glycolysis *vs* glucose oxidation *via* phosphorylation are divergent. Specifically, Th17 shows a preference for pyruvate conversion to lactate in order to expeditiously synthesize non-mitochondrial ATP (73), while Th1 cells exhibit a greater proclivity for pyruvate oxidation (20). Tregs predominantly rely on FA oxidation (FAO) and OXPHOS to maintain their function (71, 74). Memory CD4^+^ T cells primarily utilize glycolysis, while during the transition from activation to memory in CD8^+^ T cells, cellular metabolism is reprogrammed (75), leading to enhanced OXPHOS, FAO, and mitochondrial maintenance (76). As a result, memory CD8^+^ T cells primarily rely on FAO as their metabolic pathway. Overall, T cell energy metabolism includes glycolysis, FA OXPHOS, and glutaminolysis, as illustrated in **Figure 1B**.

Various enzymes and proteins participate in the metabolic processes discussed above. Among them, the glycolysis pathway is extensively and in-depth researched. The mTOR pathway and the transcription factor HIF-1α are key promoting regulatory factors in the progression of glycolysis (77, 78), while AMP-activated protein kinase (AMPK) and phosphatase and tensin homolog (PTEN) act as negative regulators (79, 80). It is worth noting that T cell surface-specific activation molecules, such as TCR (64), CD28 (81), and IL-7R (82), play important promoting roles in T cell glycolysis. In OXPHOS, FA are converted into fatty acyl-CoA in the cytoplasm and then transported into mitochondria by carnitine palmitoyltransferase I and II to play a crucial role in OXPHOS. C-Myc has an inhibitory effect on this critical enzyme. Glutaminase is a key enzyme in the glutamine metabolism process, and c-Myc has a promoting effect on glutamine metabolism (83). See **Figure 1A** for the detailed mechanisms of specific molecules and metabolic processes.

The adaptation of specific metabolic phenotypes is critical for T-cell immune function, and the regulation of cellular metabolism plays a pivotal role in shaping the plasticity of T-cell functions. Manipulating classical molecules involved in T-cell metabolism can have a profound impact on the function of these immune cells. For instance, the deficiency or restraint of mTOR can repress glycolysis, hindering the activation of CD4^+^ T cells to differentiate into Th effector cells, while promoting the generation of Tregs (84). Conversely, augmented the mammalian target of rapamycin complex 1 (mTORC1) signaling can promote Th1 or Th17 cell differentiation by enhancing glycolysis and suppressing Treg differentiation (85). Notably, although the absence of mTORC1 does not affect Treg differentiation, its function is suppressed (86–88). HIF-1α plays a crucial role in Th17 differentiation and function, while inhibiting Treg differentiation by facilitating glycolysis (72, 89). The c-Myc transcription factor mainly affects T-cell activation by modulating glycolysis. T cells deficient in c-Myc exhibit impaired proliferation and IL-2 secretion and are unable to differentiate into effector T cells (90). The AMP-activated protein kinase (AMPK) pathway is a key modulator of energy metabolism that exerts profound effects on T-cell subset differentiation. AMPK has been shown to regulate Th1 and Th17 differentiation by suppressing glycolysis through the inhibition of mTORC1. Additionally, AMPK-mediated FA OXPHOS promotes Treg and memory CD8^+^ T-cell differentiation and function, indicating the intricate interplay between metabolic pathways and T-cell plasticity (91).

## 4 T-cell metabolism-associated molecules in neuropathic pain

**Figure 2:**
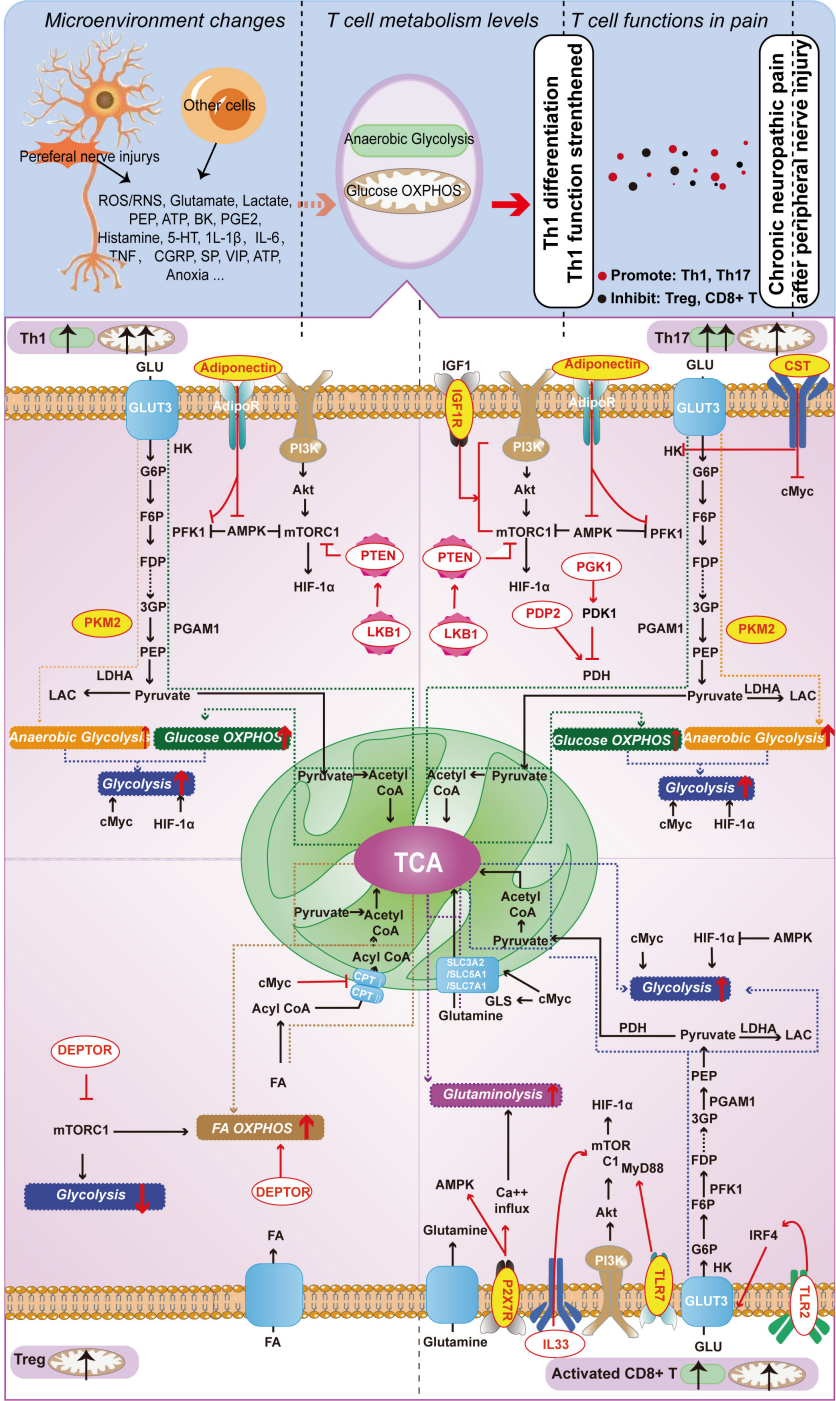
Metabolism-Related Molecules in Different T Cell Subtypes (Th1, Th17, Treg, and Activated CD8+ T Cells) Involved in Neuropathic Pain from Nerve Injury. Red letters on a yellow background indicate molecules capable of playing a role both in T cell function and in chronic neuropathic pain; red letters on a white background indicate molecules that currently play a role only in T cell function, but not in chronic neuropathic pain.

In T-cell metabolism researches, the classical molecules mentioned earlier have been thoroughly explored for their significant impact on T-cell function. We designate these molecules as the classical molecules involved in T-cell energy metabolism. However, with the advancement of research in this field, it has become apparent that certain molecules seemingly unrelated to metabolism also partake in T-cell metabolic pathways and regulate T-cell function *via* such pathways. Therefore, we classify these molecules as those that affect T-cell energy metabolism and refer to them as T-cell metabolism-related molecules. We conducted a focused screening of T-cell metabolism-related molecules in the literature over the past five years and found that these molecules do not exert their effects in every T-cell, but rather depend on specific T-cell subtypes. Subsequently, we classified T-cell metabolism-related molecules into two distinct categories based on their association with chronic neuropathic pain. The first category encompasses molecules that participate in the underlying mechanisms of chronic neuropathic pain, underscoring their importance in this condition. The second category consists of newly reported molecules that regulate T-cell metabolism, highlighting their critical role in this cellular process. Given their distinct emphases, we refer to them as “molecules affecting neuropathic pain” and “molecules affecting T-cell metabolism”, respectively. These molecules are shown in **Figure 2** and **Table 1**.

**Table 1:** Molecules of T cell energy metabolism in chronic neuropathic pain.

Molecules	Metabolism	T cell	Chronic pain (Reference)
**PKM2**	**Promotes glycolysis**	Promotes Th1 function; Promotes Th17 function.	Facilitates pain (Wang B et al., 2018)
**Adiponectin**	**Suppresses glycolysis: ↓**HIF-1α(Th1); **↓**PFK1(Th17)	Suppresses Th1 function; Suppresses Th17 function.	Alleviates pain (L Sun et al., 2018)
**IGF-1R**	**Promotes glycolysis:** ↑PI3K-AKT-mTOR signaling pathway	Promotes Th17 function.	Alleviates pain (Patrick L Stemkowski et al., 2014; Shiori Sugawara et al., 2019; Xin Chen et al., 2021)
**Cortistatin**	**Suppresses glycolysis: ↓**c-Myc; **↓**HK2.	Suppresses Th17 function	Alleviates pain (Clara P Falo et al., 2021)
**P2X7R**	**Promotes glycolysis** **Promotes mitochondrial respiration**	Maintains CD8^+^ T_M_ function;	Facilitates pain (Henrique Borges da Silva et al., 2018)
**LKB1-PTEN**	**Suppresses glycolysis:** PTEN could inhibit the mTORC1 signaling pathway, while LKB1 can promte PTEN.	Suppresses Th1 function; Suppresses Th17 function.	/
**PDP2**	**Suppresses anaerobic glycolysis:** ↑PDH	Suppresses Th17 function.	/
**PGK1**	**Promots anaerobic glycolysis:** ↑PDK1	Promotes Th17 function.	/
**DEPTOR**	**Promotes OXPHOS**	Promotes Treg function	/
**IL-33**	**Promotes glycolysis:** ↑mTORC.	Promotes CD8^+^ Tc function	/
**TLR2**	**Promotes glycolysis:** ↑IRF4; **Promotes glutaminolysis**	Promotes CD8^+^ Tc function	/

### 4.1 Molecules affecting neuropathic pain

In this section, we provide an overview of T-cell metabolism-related molecules implicated in chronic neuropathic pain over the past five years. These molecules include pyruvate kinase M2 subtype (PKM2) and adiponectin, which mainly function in Th1 cells; insulin-like growth factor 1 receptor (IGF-1R), PKM2, cortistatin (CST), and adiponectin, which primarily work in Th17 cells; and P2X receptor 7 (P2RX7) that acts in CD8^+^ T cells. We initially present how these molecules modulate the functions of distinct T cell subpopulations by regulating their energy metabolism. Subsequently, we explore the roles and underlying mechanisms of these molecules in relation to chronic neuropathic pain. Based on the discussion presented, it is clear that T cell subtypes are involved in chronic neuropathic pain (as extensively examined in the previous section), and these molecules not only affect T cell functions, but also directly influence the pathological progression of chronic pain. It implies that these molecules may affect T cell function by modulating their metabolism, ultimately contributing to the development of chronic neuropathic pain. Although direct evidence to substantiate this perspective is presently limited, the growing number of associated molecules underscores the significant potential and research value in this domain. In the subsequent sections, we will offer an in-depth analysis of these molecules.

#### 4.1.1 PKM2

Pyruvate kinase M2 subtype (PKM2), an intermediate in glycolysis, catalyzes the last step of glycolysis by the conversion from PEP to pyruvate (92). As a key enzyme in glycolysis, PKM2 is present in multiple cells and participates in metabolic processes (93, 94). The current studies demonstrate that PKM2 is requisite for Th1 and Th17 differentiation (95). Accordingly, interference with PKM2 expression by small interfering RNA (siRNA) or pharmacological inhibition of PKM2 activity could suppress its involvement in glycolysis and subsequently restrict the function of Th1 and Th17 (96, 97). It has been demonstrated that PKM2 is a potential therapeutic target in various diseases (98–100). For instance, in a recent study, PD-1-targeted particles suppressed activated T cells and alleviated autoimmunity exactly *via* inhibition of PKM2-mediated glycolysis (98).

The role of PMK2 in neuropathic pain has been studied (101). In the rat animal model of CCI-induced neuropathic pain, peripheral nerve injury significantly raised PKM2 levels in spinal cord. Double immunofluorescence staining displayed co-localization of PKM2 with neurons, microglia and astrocytes. Intrathecal injection of PKM2 siRNA leads to attenuation of CCI-induced extracellular regulated protein kinases (ERK) and signal transducer and activator of transcription 3 (STAT3) activation, as well as CCI-induced mechanical allodynia and thermal hyperalgesia. The above findings suggest that inhibition of PKM2 expression can effectively attenuate CCI-induced neuropathic pain and inflammatory responses in rats, possibly through modulation of ERK and STAT3 signaling pathways (101). This study illustrates that PKM2 is an important contributor to neuropathic pain, however it fails to focus on the impact on T cell metabolism in chronic pain, which would be a valuable direction for future research.

#### 4.1.2 Adiponectin

Adiponectin is an adipocytokine primarily secreted by adipose tissue (102), modulating the regulation of glucose and FA oxidation (103, 104). Adiponectin binds to several receptors, including two adiponectin receptors (AdipoR1 and AdipoR2) and one cadherin-like receptor (105). Previous studies have demonstrated that Adiponectin exerts a pivotal regulatory influence on T-cell differentiation and function (106). Recent research has revealed that in Th1, after binding to the adiponectin receptor, adiponectin can interfere with the transcription factor HIF-1α, resulting in the suppression of glycolysis. Similarly, upon binding to AdipoR1, Adiponectin can suppress phosphofructokinase 1 (PFK1), the principal enzyme involved in glycolysis, thereby influencing glycolysis and inhibiting Th17 function (107, 108). These findings suggest that adiponectin can modulate T-cell metabolism to affect its function, thereby suggesting the potential of adiponectin as a therapeutic target for T-cell-related diseases. Notably, in patients with obesity, adiponectin can regulate the activity of pro-inflammatory CD4^+^ T cells (108), and its effectiveness in treating obesity has been demonstrated (109).

Studies have demonstrated that adiponectin plays a protective role in neuropathic pain (110). L Sun and colleagues conducted experiments on wild-type (WT) and adiponectin-knockout (KO) mice with partial sciatic nerve ligation (pSNL) or sham surgery, assessing pain behavior and protein levels (110). The results revealed that adiponectin-KO mice exhibited significantly lower thermal and mechanical pain thresholds than WT mice under both physiological and pathological conditions. Adiponectin was found to regulate thermal nociception by inhibiting the activation of p38 MAPK and transient receptor potential cation channel subfamily V member 1 (TRPV1) in neurons, microglia, and cortical neurons, underscoring its regulatory role in neuropathic pain. Targeting adiponectin may be a promising therapeutic strategy for reducing thermal sensitivity *via* inhibiting the activation of DRG neurons, spinal cord microglia, and somatosensory cortical neurons. In addition, adiponectin has been shown to regulate T-cell metabolism and function. Targeting Th1 and Th17 metabolism may inhibit the function of these T cells, which could alleviate neuropathic pain. Although further research is needed to fully explore this treatment approach, it offers a novel direction for developing more effective neuropathic pain treatment strategies.

#### 4.1.3 IGF-1R

The IGF system is essential in diverse physiological processes, including cellular metabolism, growth, and differentiation (111). Insulin-like growth factor 1 receptor (IGF-1R) is one of the major signaling receptor tyrosine kinases that mediate the actions of IGF1 and IGF2 (112), which could be expressed in CD4^+^ T cells, particularly the Th17 (113). One step further research indicates, in CD4^+^ T cells, IGF1 binding to IGF1R mediates signaling through the PI3K-AKT-mTOR pathway to increase anaerobic glycolysis, which subsequently facilitates Th17 cell differentiation (113). Thus, IGF1 might be considered a decision-making molecule for Th17 differentiation, on the basis of an enhanced pathway of anaerobic glycolysis. This decisional role makes its expression on T cells necessary for the full development of pathogenic Th17-mediated CNS inflammation in EAE, and a potential therapeutic target candidate for new therapies to treat autoimmune diseases. Lenaldekar (LDK), a novel small molecule targeting the IGF-1R signaling pathway, has been studied to reduce T cell proliferation and attenuate disease-related clinical symptoms (114).

Several studies have long focused on IGF1 as a factor contributing to neuropathic pain (115, 116). Following peripheral nerve injury, impaired neurons release this molecule into the synaptic environment, acting on IGF1R on DRG neurons and consequently mediating an enhancement of protein kinase C alpha (PKCα)-dependent T-type calcium currents to increase pain. Interfering with this pathway could potentially reduce mechanical and thermal pain hypersensitivity in rodents (115). A recent study demonstrated that IGF1 is not only produced by cultured neurons, but also by astrocytes (117), while IGF1R is primarily expressed in neurons (118). It focused on the study that IGF1 acting on neurons could promote neuropathic pain by mediating mTOR-related signaling, with intrathecal injection of IGF1R inhibitors or IGF1-neutralizing antibodies attenuating CCI-induced pain behavior (118). Of note, in the previous studies, IGF1R is also expressed in T cells in the CNS (113); thus, the T-cell metabolic pathway mediated by IGF1R is of great potential research value in neuropathic pain.

#### 4.1.4 Cortistatin

Cortistatin (CST) is a cyclic neuropeptide with a Cys-Cys ring, which has a strong inhibitory effect on cortical neurons (119) and immune cells (120). In recent years, CST has been found to have immunomodulatory effects in various disease models. For instance, in a rat model of collagen-induced arthritis, berberine can induce CST in the gut to inhibit Th17 cell response and ameliorate arthritis symptoms (121). This suggests that CST plays a crucial role in the regulation of Th17 cells, but its inhibitory mechanism remains poorly explored. To address this issue, Guo et al. conducted a study aimed at elucidating the underlying mechanisms of CST in Th17 (122). It revealed that CST exerts its inhibitory effect on Th17 differentiation by regulating glycolysis. More specifically, CST was found to significantly suppress the glycolytic activity of Th17, while downregulating the mRNA expression of two key glycolytic molecules, namely c-Myc and HK2. Notably, overexpression of c-Myc and HK2 almost completely abolished the inhibitory effect of CST on Th17 cell differentiation, highlighting the critical involvement of the c-Myc-HK2 pathway in the CST-mediated inhibition of Th17 differentiation. Furthermore, the study identified the growth hormone secretagogue receptor 1 (GHSR1) as the mediator of the inhibitory effect of CST on Th17 differentiation. These findings provide important insights into the mechanism of CST in immune regulation and shed light on a novel therapeutic target for the treatment of immune-related disorders.

Correspondingly, amongst studies on neuropathic pain, Mario Delgado’s team proposed in 2014 that CST, a natural analgesic component of the peripheral injury receptor system, produced by peptidergic injury receptor neurons in the DRG in response to inflammation and noxious stimuli (123), plays an essential pain suppressing role in persistent inflammatory pain. Recently, Mario Delgado’s team presented new evidence on the role of CST in neuropathic pain (124). They found that the injection of CST, both peripherally and centrally, was able to alleviate the hyperalgesia and allodynia associated with peripheral nerve injury and diabetic neuropathy. The analgesic effect of CST targeted multiple aspects, regulating hypersensitization of nociceptors, inhibiting neuroinflammatory responses, and enhancing production of neurotrophic factors. Deficiency in CST worsened neuropathic pain responses and peripheral nerve dysfunction. These findings suggest that CST-based treatments may offer an appealing substitute for managing chronic neuropathic pain, and the multi-pronged analgesic effects of CST hint at the possibility of T cell energy metabolism as a forthcoming therapeutic target.

#### 4.1.5 P2RX7

Extracellular ATP (eATP) is a “danger signal” used by eukaryotes to detect cell damage (125). In mice and humans, the release of eATP during inflammation or injury stimulates innate immune activation and neuropathic pain *via* the purinergic receptor P2X receptor 7 (P2RX7) (126–128). Notably, P2RX7 was expressed in diverse cells and was capable of facilitating both glycolysis (129, 130) and mitochondrial respiration (131). Nevertheless, the regulation of energy metabolism suggests that P2RX7 may be instrumental in leaning immune cell differentiation, which is likely due to the fact that P2RX7 activity may be governed by multiple factors. Therefore, under different circumstances, P2RX7 may predominantly support one metabolic pathway (e.g. respiration) over another (e.g. glycolysis). For example, in mice, eATP and P2RX7 have been shown to be requisite for Th17 differentiation (132). This process could be enhanced by glucose metabolism, which might be facilitated by lower sensitivity to mitochondrial damage, or by the preferential coupling of P2RX7 and glycolysis in this cell type. Recently, it was reported that P2RX7 promoted mitochondrial homeostasis and metabolic function in differentiating memory CD8^+^ T cells (133). It also showed that P2RX7 is required for the establishment, maintenance and function of long-lived central and tissue-resident memory CD8^+^ T cell populations in mice. In contrast, P2RX7 is not required for the generation of short-lived effector CD8^+^ T cells. Specifically, in activated CD8^+^ T cells, P2RX7 was stimulated by eATP (derived from damaged cells or exported from activated living cells). Calcium influx was subsequently induced, increasing mitochondrial metabolic activity (133), which can be reflected in enhanced glutaminolysis and enhanced FA OXPHOS. It is important to emphasize that memory CD8^+^ T cells function primarily in the metabolic mode of FA OXPHOS, whereas activated CD8^+^ T cells function primarily in the metabolic modes of glycolysis and glutaminolysis. Therefore, we speculate that differences in the primary metabolic modality used may contribute to the different roles of P2RX7 in the importance of memory CD8^+^ T cells and activated CD8^+^ T cells.

Previous studies have revealed that P2RX7 regulates metabolic processes, for which it is considered as a promising pharmacological target for the treatment of neuropathic pain (132, 134, 135). In a recent study, P2RX7 can guide the metabolic adaptation of long-lived memory CD8^+^T cells. At the same time, we also observed that the pharmacological inhibitor of P2RX7 stimulated the metabolism and differentiation disorder of activated mouse and human CD8^+^ T cells *in vitro*. *In vivo*, the transient blockage of P2RX7 improved neuropathic pain, but also impaired the production of memory CD8^+^ T cells (133). It suggests that the mechanism underlying the amelioration of neuropathic pain with suppression of P2RX7 is, at least in part, through inhibiting active CD8^+^ T cell metabolism to consequently affect the role of active CD8^+^ T cells in chronic neuropathic pain. Furthermore, besides enhancing mitochondrial stability, AMPK (which inhibits HIF-1α and thus glycolysis) is induced by this kind of T-cell metabolic inhibition. Remarkably, the role of memory CD8^+^ T cells in neuropathic pain has been neglected and little studied in previous studies. Our focus here is also on the effect of P2RX7 on active CD8^+^ T metabolism. Thus, the present study also provides a different perspective to examine the role of memory CD8^+^ T cells in neuropathic pain.

### 4.2 Molecules affecting T-cell metabolism

Regarding the molecules that have not been implicated in chronic pain, we concentrate on elucidating their function and specific mechanisms in regulating T-cell metabolism. These molecules include liver kinase B1-phosphatase and tension homolog deleted on chromosome 10 (LKB1-PTEN), which primarily function in Th1 cells; pyruvate dehydrogenase phosphatase catalytic subunit 2 (PDP2), phosphoglycerate kinase 1 (PGK1), and LKB1-PTEN, which primarily function in Th17 cells; DEP domain containing mTOR interacting protein (DEPTOR), which has a significant impact on Treg function; interleukin-33 (IL-33) and Toll-like receptor 2 (TLR2), which are critical participants in CD8^+^ T cell responses. Despite the lack of demonstrated involvement in chronic neuropathic pain treatment, these molecules hold the potential to emerge as targets in this field, rendering them of significant interest for comprehensive reviews. It should be noted that, in fact, there are additional molecules beyond those presented above. Due to the limitations in space, we have compiled a comprehensive list of these unmentioned molecules in **Table S1**.

#### 4.2.1 LKB1-PTEN

The mTORC is a central regulator of T cell metabolic reprogramming, with the liver kinase B1-phosphatase and tension homolog deleted on chromosome 10 (LKB1-PTEN) signaling pathway being one of its upstream regulators. LKB1 and PTEN directly interact with each other, and LKB1 can phosphorylate PTEN to inhibit the mTORC1 signaling pathway, independent of AMPK. Consequently, LKB1 deficiency results in increased mTORC1 activity and upregulation of glycolysis mediated by HIF-1α. Further experiments have shown that Th17 and Th1 cell bias in LKB1-deficient T cells is mediated by glycolysis. These findings highlight the critical role of the LKB1-PTEN signaling pathway in regulating T cell metabolism and immune balance. Through the modulation of glycolysis, the LKB1-PTEN signaling pathway regulates the differentiation of Th1 and Th17 cells, thereby maintaining the internal homeostasis and immune balance of T cells (136).

#### 4.2.2 PDP2

Recent studies have identified pyruvate dehydrogenase phosphatase catalytic subunit 2 (PDP2) as a crucial regulator of T cell metabolism, specifically in the glycolysis and glucose OXPHOS pathways (137). While expressed in both Th1 and Th17 cells, PDP2 plays a role in restricting Th17 differentiation by inhibiting glycolysis. This function is closely associated with pyruvate dehydrogenase (PDH). PDH is a critical branching enzyme that converts pyruvate to acetyl-CoA, which is then transported to the mitochondria for OXPHOS. Due to the advantage of Th17 cells to convert pyruvate to lactate for rapid generation of non-mitochondrial ATP, PDH has been shown to uniquely regulate Th17 cells (73). Further, PDP2 has been identified as a key factor that promotes PDH activity *in vivo*, facilitating glucose metabolism and the subsequent steps of glycolysis and glucose OXPHOS. Taken together, these findings emphasize the critical role of PDP2 in regulating T cell metabolism and function, with a particular focus on Th17.

#### 4.2.3 PGK1

Phosphoglycerate kinase 1 (PGK1) plays a crucial role in metabolic regulation in CD4^+^ T cells. Recently, Yang Lu et al. found that both CD4^+^ T cells and Th17 exhibited increased glycolysis and PGK1 expression in the hearts of mice with myocarditis (138). Treatment of mice with the PGK1 inhibitor NG52 resulted in reduced inflammation and fibrosis in the heart, improved cardiac contractile function, reduced infiltration of Th17 and Th1 in the heart, and increased Treg proportion. In addition, NG52 could inhibit the activation and differentiation of CD4^+^ T cells from mice with myocarditis and patients with myocarditis *in vitro*. The mechanism underlying the effects of the PGK1 inhibitor involves the suppression of glycolytic activity and the reduction of the phosphorylation of pyruvate dehydrogenase kinase 1 (PDHK1), leading to increased production of ROS in the mitochondria, which in turn inhibits Th17 differentiation. Previous studies have shown that PDHK1 is a specific enzyme in Th17 that is almost not expressed in Th1 or other T-cell subsets. PDHK1 can inhibit PDH, which suppresses pyruvate entry into mitochondria and promotes Th17 glycolysis (73). In summary, PGK1 plays a metabolic regulatory role in CD4^+^ T cells and regulates the function of Th17 by modulating their glycolytic metabolism.

#### 4.2.4 DEPTOR

DEP domain containing mTOR interacting protein (DEPTOR) is an evolutionarily conserved intracellular binding partner of mTOR and serves as a negative regulator of signal transduction. In this study, we identified the expression of DEPTOR in CD4^+^ T cells and observed that its relative expression levels modulate differentiation and glucose utilization of CD4^+^ T effector cells *in vitro*. Using a knock-in mouse model, we further found that induced expression of DEPTOR in CD4 T regulatory cells stabilizes Foxp3 expression, promotes a shift towards OXPHOS metabolism, and enhances survival and suppressive function. These findings demonstrate the critical regulatory role of DEPTOR in CD4^+^ T cells, particularly in Tregs, and highlight the close interplay between this process and T cell metabolism (139).

#### 4.2.5 IL-33

Interleukin-33 (IL-33), a crucial member of the IL family, plays a crucial role in both innate and adaptive immunity. Recent research has revealed that IL-33 induces activation and proliferation of CD8^+^ T effector cells through extracellular signaling pathways, rather than nuclear signaling pathways (140). Specifically, IL-33 activates the mTORC1 pathway, enhancing glucose uptake and lactate production in CD8^+^ T cells, thereby promoting accelerated anaerobic glycolysis and increased activation of effector T cells. This discovery not only sheds light on the impact of IL-33 on CD8^+^ T cell function and underlying metabolic mechanisms but may also have profound implications for the future treatment strategies of CD8^+^ T cell related diseases.

#### 4.2.6 TLR2

Recent research has found that Toll-like receptor 2 (TLR2), a co-stimulatory molecule, plays a role in enhancing TCR-dependent activation of CD8^+^ T cells when activated (141). Specifically, TLR2 agonist Pam3CSK4 was shown to directly enhance TCR-dependent activation of CD8^+^ T cells (141). Transcriptome analysis further revealed that TLR2 signaling increases the expression of genes related to the cellular energy metabolism of CD8^+^ T cells, such as Interferon Regulatory Factor 4 (IRF4), leading to improved glycolysis and glutaminolysis. This effect can further increase the expression of genes related to immune regulation and function, such as IFN-γ. The metabolic processes of glycolysis and glutaminolysis are necessary for the enhanced T cell activation mediated by TLR2. Overall, TLR2 promotes CD8^+^ T cell immune response and function by regulating their energy metabolism.

## 5 Discussion

Chronic neuropathic pain is a complex and multifaceted condition that arises from pathological changes or disorders within the nervous system (142). It can be classified as either central or peripheral neuropathic pain (143), with the latter having relatively well-established researches compared to the former (144, 145). Peripheral neuropathic pain can be caused by a multitude of factors, including peripheral nerve injury, chemotherapy, and nerve inflammation-induced neuropathic pain (146). In this review, we focus on neuropathic pain resulting from peripheral nerve injury, which has been extensively studied in animal models (144, 145) and has well-established mechanisms involving both peripheral and central sensitization.

Numerous studies have explored the energy metabolism of T cells in autoimmune and inflammatory disorders, including but not limited to rheumatoid arthritis (147) and systemic lupus erythematosus (20, 148). In these diseases, pro-inflammatory T cells, including Th1 and Th17 cells (particularly Th17), play a crucial role and rely primarily on glycolysis for their functions (149, 150). Therefore, most researches on T cell metabolism have focused on the glycolytic pathway of Th17 cells, which explains why this review is more detailed in exploring the molecular mechanisms of Th17 glycolysis. It should be emphasized that T cell energy metabolism in cancer has also been extensively studied. Nonetheless, these studies have been excluded from this review due to the distinct energy metabolism of T cells within the tumor microenvironment compared to that of T cells in physiological conditions or other diseases (151).

Previous studies on neuropathic pain resulting from peripheral injuries have identified the release of a series of mediators during nerve damage. This event leads to the infiltration of other inflammatory and immune cells in the area, which then continue to release substances and ultimately form a microenvironment that can profoundly impact the cells present and cause metabolic abnormalities (45). Notably, metabolic reprogramming of glial cells has been reported in chronic neuropathic pain (41). Such conditions may also occur in T cells. This review places particular emphasis on the molecular aspects of T cell energy metabolism and explores its current involvement in neuropathic pain. By establishing a link between T cell metabolism and neuropathic pain resulting from peripheral injuries, this review provides a potential target for intervening in neuropathic pain.

During nerve injury, the release of eATP can activate P2RX7 on CD8^+^ T cells, leading to mitochondrial homeostasis and affecting energy metabolism pathways, including lipid OXPHOS and glutamine catabolism, which ultimately influence the function of CD8^+^ T cells. Notably, P2RX7 is essential for the differentiation of memory CD8^+^ T cells. Moreover, pharmacological inhibition of P2RX7 *in vitro* induced metabolic and differentiation dysregulation in activated mouse and human CD8^+^ T cells, while transient P2RX7 blockade improved neuropathic pain *in vivo* but impaired memory CD8^+^ T cell production. Hence, in chronic neuropathic pain, P2RX7 can modulate T cell function through alterations in energy metabolism, while blockade of P2RX7 can ameliorate neuropathic pain (133). This study provides a cogent link between neuropathic pain and T cell metabolism, yet the contribution of memory CD8^+^ T cells in neuropathic pain requires further elucidation. While direct evidence connecting T-cell metabolism and neuropathic pain is limited, this review takes a novel approach by exploring how molecules that impact T-cell function by affecting T-cell energy metabolism and how these molecules can contribute to chronic neuropathic pain. which bridges the connection between T-cell energy metabolism and neuropathic pain. Finally, this review explores some T-cell energy metabolism-associated molecules that have not yet been explored in neuropathic pain. These molecules may represent potential targets for the treatment of neuropathic pain, and the exploration of T cell energy metabolism as a novel avenue for neuropathic pain therapy is an area ripe for further development.

It is essential to highlight that this review was manually searched for relevant studies. Although we have categorized the research according to a certain logic, it still cannot cover all the pertinent studies. We refer to the molecules that play a role in T-cell energy metabolism as T-cell energy metabolism-related molecules and categorize them into molecules that act in neuropathic pain and those that have not yet been found to act in neuropathic pain. Furthermore, we set our screening criteria to new molecules related to T cell metabolism within the last five years to gather as comprehensive a collection of related studies as feasible. It is crucial to note that this article can also serve as a point of reference for exploring other types of neuropathic pain. For instance, the key transcription factor HIF-1α in T cell glycolysis plays a vital role in neuropathic pain. In the initial stages of the CRPS mouse model, inhibiting HIF-1α can produce an anti-abnormal pain effect and suppress the production of inflammatory cytokines (152). Hyaluronic acid (HA)/CD44 regulates Th1 differentiation by activating the Annexin A1-AKT-mTOR signaling pathway, promoting the pathogenesis of chronic prostatitis/chronic pelvic pain syndrome (CP/CPPS). AKT is involved, which may be linked to its regulation of glycolysis (153). However, the changes in T-cell energy metabolism in the corresponding disease environment must be taken into account. To conclude, exploring the connection between T cell energy metabolism and neuropathic pain is a research area that merits in-depth exploration.

## 6 Conclusion and perspective

Neuropathic pain, resulting from peripheral nerve damage, leads to microenvironmental changes due to the buildup of substances such as inflammatory cytokines and metabolic byproducts. These changes may influence cellular metabolism and function, thus altering their involvement in chronic pain, as evidenced in glial cells (41). This review intends to investigate the relationship between T-cell metabolic alterations, their function, and their contribution to chronic pain. Firstly, we elucidate the impact of metabolic pathway alterations on T-cell function to link T cell metabolism and function. Subsequently, we investigate how microenvironmental alterations lead to T-cell metabolic changes, connecting chronic pain and T-cell metabolism. Lastly, we focus on recent advances in molecules related to T-cell metabolism over the past five years. Some molecules play pivotal roles in both T-cell metabolism and chronic pain. We clarify how they modulate distinct T-cell subset functions by regulating energy metabolism and further elucidate their roles and potential mechanisms in chronic neuropathic pain. This implies that these molecules can influence T-cell function through metabolic alterations, thus promoting chronic neuropathic pain development. Despite limited direct evidence, the growing number of associated molecules underscores the field’s significant potential and research value. For other molecules not yet linked to chronic pain, we focus on their specific mechanisms and roles in T-cell metabolic regulation. Although not currently employed in the treatment of chronic pain, these molecules show promise as potential targets.

Thus, in future research, comprehensively exploring T-cell metabolism in chronic pain may enhance our understanding of T-cell mechanisms in chronic pain and establish a theoretical basis for novel therapies. Moreover, attention should be focused on T-cell metabolic molecules that have yet to be explored in chronic pain, as they could hold significant roles and potentially serve as future targets for chronic pain treatment. However, the complexity of chronic pain, involving multiple pain types and corresponding microenvironmental changes, along with the intricate nature of T-cell metabolism processes, including numerous metabolic pathways, interactions, and regulations, render the exploration of T-cell metabolism in chronic pain more challenging. Despite these challenges, this research field offers promising prospects and merits further exploration. In summary, delving deeper into T-cell metabolism in chronic pain may offer new insights and therapeutic strategies to address this complex pathological issue in chronic pain.

## Author contributions

All authors made a significant contribution to the work reported, whether that is in the conception, study design, execution, acquisition of data, analysis and interpretation, or in all these areas; XD took part in drafting, revising or critically reviewing the article; RC and JY gave final approval of the version to be published; MD and JL have agreed on the journal to which the article has been submitted; and YL and SS agree to be accountable for all aspects of the work.
