# Supplementary material for: The potential role of T-cell metabolism-related molecules in chronic neuropathic pain after nerve injury: a narrative review
Source: Front Immunol. 2023 May 17;14:1107298. doi: 10.3389/fimmu.2023.1107298 (PMC10229812; doi:10.3389/fimmu.2023.1107298)
Supplement: Supplementary file 1 [file Table_1.docx]

**Table S1 Molecules of T cell energy metabolism within 5 years.**

| **Molecules** | **Metabolism** | **T cell** | **Reference(PMID)** |
| --- | --- | --- | --- |
| **LKB1-PTEN** | **Suppresses glycolysis:** PTEN could inhibit the mTORC1 signaling pathway, while LKB1 can promote PTEN. | Suppresses Th1 function; Suppresses Th17 function. | 34003330 |
| **Iron** | **Promotes glycolysis:** promote AKT-mTOR signaling pathway | Promotes Th1 function.  Promotes Th17 function. | 35917405 |
| **PDP2** | **Suppresses anaerobic glycolysis:** ↑PDH | Suppresses Th17 function. | 30150402 |
| **PGK1** | **Promotes anaerobic glycolysis:** ↑PDK1 | Promotes Th17 function. | 36726197 |
| **Glutaminase 1 (Gls1)** | **Promotes glycolysis:** ↑HIF-1α. | Promotes Th17 function. | 31233276 |
| **Bcl-3** | **Promotes glycolysis** | Promotes Th17 function. | 36159779 |
| **AdipoR1** | **Promotes glycolysis:** ↑HIF-1α. | Promotes Th17 function. | 32973810 |
|  |  |  |  |
| **PEP** | **Promotes glycolysis** | Promotes Th17 function. | 36857180 |
| **DEPTOR** | **Promotes OXPHOS** | Promotes Treg function | 29969188 |
| **KLF10** | **Promotes glycolysis**  **Promotes mitochondrial respiration** | Promotes Treg function | 33378664 |
| **Oleic acid** | **Promotes FA OXPHOS** | Promotes Treg function | 33170805 |
| **IL-33** | **Promotes glycolysis:** ↑mTORC. | Promotes CD8+ Tc function | 34411293 |
| **TLR2** | **Promotes glycolysis:** ↑IRF4;  **Promotes glutaminolysis** | Promotes CD8+ Tc function | 31636238 |
